# Commentary: Wuling powder ameliorates diarrhea-predominant irritable bowel syndrome in mice by modulating gut mucosal microbiota and alleviating intestinal inflammation

**DOI:** 10.3389/fcimb.2025.1694104

**Published:** 2025-09-30

**Authors:** Kangxiao Guo, Junping Zou, Yuan Tang

**Affiliations:** Changsha Health Vocational College, Changsha, China

**Keywords:** diarrhea-predominant irritable bowel syndrome, Wuling powder, gut mucosal microbiota, inflammatory factors, gut microbiota metabolites, gut-kidney axis, intestinal mucosal barrier

## Introduction

Diarrhea-predominant irritable bowel syndrome (IBS-D) is a highly prevalent functional gastrointestinal disorder, primarily characterized by recurrent diarrhea, abdominal pain, and visceral hypersensitivity. Clinically, the disease follows a chronic relapsing course, which severely impairs patients’ quality of life (Li et al., 2016). Traditional Chinese Medicine (TCM) formulas are widely used in the treatment of IBS-D, and their advantages of multi-targeted and multi-pathway regulation are consistent with the complex pathogenesis of IBS-D. From the perspective of TCM theory, the occurrence of IBS-D is closely associated with the liver-spleen disharmony syndrome which emotional disorders lead to liver qi stagnation, while spleen-stomach weakness impairs digestive and absorptive functions, the combined effect of these two factors triggers gut dysfunction (Liu et al., 2021; Shen et al., 2021). Wuling Powder, derived from *Treatise on Febrile Diseases*, is commonly used clinically for treating gastrointestinal diseases related to the Water-Dampness syndrome. Existing studies have indicated that gut microbiota imbalance and gut inflammation are the core pathological drivers of IBS-D (Yuan et al., 2023). As a key component of the gut microecosystem, the gut mucosal microbiota has direct interactions with the gut mucosal barrier and the immune system: its imbalance disrupts barrier integrity, triggers pro-inflammatory responses, and exacerbates IBS-D symptoms (Fusco et al., 2023; Hays et al., 2024). In addition, the imbalance of the gut-kidney axis is also associated with functional gastrointestinal disorders: gut inflammation and toxins secreted by the microbiota can translocate to the kidneys, inducing or exacerbating kidney injury, which in turn further aggravates gut dysfunction (Li et al., 2024; Shen et al., 2025).

Recently, Tian et al. (2025) published an original research paper in *Frontiers in Cellular and Infection Microbiology*, which systematically explored the therapeutic effect of Wuling Powder on IBS-D model mice and its potential mechanism of action-exerting curative effects by regulating the gut mucosal microbiota and alleviating gut inflammation. This commentary aims to summarize the core findings of this study, analyze its methodological advantages, identify current limitations, and propose targeted directions for in-depth research in the future. Ultimately, it intends to provide references for deepening the understanding of the anti-IBS-D mechanism of Wuling Powder and promoting its clinical translation.

## General comments

Tian’s research focused on the therapeutic potential of Wuling Powder for IBS-D, with a key exploration of its regulatory effects on the structure of gut mucosal microbiota, inflammatory responses, and gut barrier function. The study evaluated the intervention effects from multiple dimensions, including general clinical symptoms, the structure and diversity of gut mucosal microbiota, the levels of inflammatory factors, and the correlation between microbiota and inflammatory factors. The results showed that Wuling Powder significantly improved IBS-D symptoms, reduced watery stools, and increased the activity of mice. It also restored the diversity of gut mucosal microbiota and exerted targeted regulation on characteristic bacterial genera. In the treatment of IBS-D, Wuling Powder decreased the levels of pro-inflammatory cytokines. Correlation analysis confirmed that *Sporosarcina* was positively correlated with MFGE8, and *Paludibaculum* was negatively correlated with both TNF-α and IL-6. Based on these findings, the research team concluded that Wuling Powder improves IBS-D primarily by regulating gut mucosal microbiota and alleviating gut inflammation.

Tian et al.’s study demonstrates significant advantages in experimental design, outcome assessment, and mechanism exploration, which enhance the scientific rigor and translational value of the research. First, the model design is clinically relevant. Unlike single-factor models, the combined use of *Sennae Folium* and restraint-tail clamping stress not only recapitulates the core clinical symptoms of IBS-D but also simulates its TCM syndrome. This model reflects the psychophysiological characteristics of IBS-D, making the experimental results more translatable to clinical practice. Second, the methodology is systematic and multi-dimensional. The study integrated gut mucosal microbiota analysis, inflammatory factor detection, and correlation analysis, constructing a complete evidence chain for “microbiota dysbiosis- intestinal inflammation-IBS-D symptoms. “Additionally, the administration of Wuling Powder in decoction form aligns with clinical TCM medication practices, ensuring high bioavailability and enhancing translational potential. Third, the study fills gaps in TCM microecological research. By identifying *Sporosarcina* and *Paludibaculum* as potential microbial biomarkers for IBS-D, it provides novel microecological diagnostic indicators for TCM treatment of IBS-D. Moreover, clarifying the direct association between characteristic microbiota and inflammatory factors not only elucidates the mechanistic basis of Wuling Powder but also offers a new framework for understanding the gut microbiota-immune system interaction in IBS-D.

Despite its significant value, this study has the following limitations that need to be further addressed in future research. The study only verified the efficacy of the full-decoction of Wuling Powder, without identifying the specific chemical components that mediate microbiota regulation and anti-inflammatory effects. As a multi-component TCM formula, Wuling Powder contains compounds such as pachyman, atractylenolide III, and alisol A, the individual effects of these components have not yet been clarified. gut microbiota exerts physiological functions mainly through metabolites. This study did not detect such metabolites, which limits the understanding of the “microbiota-metabolite-host” interaction. For instance, SCFAs are key anti-inflammatory metabolites produced by gut bacteria, while TMAO is associated with gut barrier damage; the roles of both in the treatment of IBS-D with Wuling Powder remain unclear. Recent studies have confirmed that IBS-D-related gut inflammation can induce kidney injury through the intestine-kidney axis (Li et al., 2024). However, this study did not explore whether Wuling Powder has multi-organ regulatory effects, which limits the understanding of its systemic therapeutic potential. In addition, although the study mentioned the barrier repair factor MFGE8, it did not investigate the effect of Wuling Powder on the structural integrity of the gut mucosal barrier, nor did it study its interaction with mucosal immune cells. This results in an insufficient understanding of the mechanism by which Wuling Powder protects the gut barrier.

In order to fully clarify the therapeutic mechanism of Wuling Powder and facilitate its clinical translation, further research is required in the following aspects. Chromatographic techniques such as High-Performance Liquid Chromatography (HPLC) and Ultra-High Performance Liquid Chromatography-Mass Spectrometry (UPLC-MS) should be used to separate and identify the key active monomers in Wuling Powder. *In vitro* experiments need to be conducted to verify the effects of these monomers on the expression of characteristic bacterial genera and inflammatory factors. Combined with Pharmacokinetics-Pharmacodynamics (PK-PD) correlation analysis, it is necessary to clarify whether the active ingredients exert their effects directly in the intestine or indirectly through systemic circulation. Gas Chromatography-Mass Spectrometry (GC-MS) should be employed to detect the levels of short-chain fatty acids (SCFAs) in the gut lumen and serum of IBS-D mice before and after Wuling Powder intervention. Meanwhile, 16S rRNA sequencing should be combined to analyze whether Wuling Powder exerts its effects by promoting the proliferation of SCFA-producing bacteria and regulating the SCFA synthesis pathway. Additionally, the impact of Wuling Powder on harmful metabolites, such as TMAO, should be evaluated to determine if it improves IBS-D by balancing beneficial and harmful metabolites. An IBS-D combined with renal injury model should be established to simulate the clinical situation of comorbidity between IBS-D and Chronic Kidney Disease. By detecting renal function indicators and pathological changes in kidney tissue, the multi-organ protective effect of Wuling Powder can be verified. Furthermore, gut-derived toxins and differentially expressed genes in the kidney, Transforming Growth Factor should be analyzed to clarify the intestine-kidney communication pathway mediated by Wuling Powder (Wei et al., 2024). Techniques including Immunohistochemistry, Western Blot, and Quantitative Real-Time Polymerase Chain Reaction should be used to detect the expression of tight junction proteins and Mucin 2 in the gut mucosa. By isolating gut mucosal immune cells, it is necessary to explore whether the microbiota (or their metabolites) regulated by Wuling Powder affect the activation of immune cells, thereby clarifying the “microbiota-immune cell-barrier” regulatory network.

## Discussion

In conclusion, the study by Tian et al. has laid a solid foundation for understanding the microecological mechanism of Wuling Powder in the treatment of IBS-D. The key findings of this study—including the regulatory effect of Wuling Powder on gut mucosal microbiota (e.g., restoring diversity, targeting Sporosarcina and Paludibaculum), its inhibitory role in pro-inflammatory cytokines, and the validity of the clinically relevant IBS-D mouse model—provide important preliminary evidence for the clinical application of Wuling Powder. From a clinical perspective, these mechanistic insights could directly inform the design of future clinical trials: for example, by using Sporosarcina and Paludibaculum as potential diagnostic or prognostic biomarkers, researchers can stratify IBS-D patients more accurately, enabling enrollment of subgroups most likely to benefit from Wuling Powder treatment and thereby improving trial efficiency and success rates. Additionally, the confirmation that Wuling Powder’s decoction form (consistent with clinical TCM practice) exerts therapeutic effects supports its direct translation to clinical settings, while future identification of active components could facilitate the development of standardized preparations—addressing issues of batch-to-batch variability in traditional decoctions and promoting the adoption of Wuling Powder in evidence-based clinical practice for personalized treatment. For instance, if specific monomers (e.g., pachyman, atractylenolide III) are confirmed to mediate the regulation of SCFA-producing bacteria or inhibition of pro-inflammatory factors, they could serve as quality control markers for Wuling Powder preparations, ensuring consistent efficacy across different clinical applications.

Moreover, the limitations identified in Tian et al.’s study (e.g., lack of active component identification, unaddressed gut-kidney axis, insufficient metabolite analysis) align with critical gaps in current TCM research on gastrointestinal disorders. Traditional TCM research often focuses on the overall efficacy of multi-component formulas but lacks clarity on “active ingredient–target” relationships, which hinders the integration of TCM with modern pharmacology and international recognition. The proposed future directions—such as using UPLC-MS for active monomer isolation and GC-MS for metabolite profiling—directly address this gap by applying modern analytical techniques to dissect the complex composition of Wuling Powder, bridging the gap between TCM’s holistic approach and modern molecular pharmacology. Similarly, exploring the gut-kidney axis expands the understanding of TCM’s “multi-organ regulation” concept (e.g., the TCM theory of “kidney governing water metabolism and spleen governing transportation and transformation” interacting to affect gastrointestinal function) into a mechanistic framework (e.g., gut-derived toxins mediating kidney injury and subsequent gut dysfunction), thereby enriching the theoretical system of TCM in treating IBS-D.

Future in-depth studies focusing on active ingredients, metabolites, the intestine-kidney axis, and the mucosal barrier will not only enrich the theoretical system of TCM in treating IBS-D but also provide a scientific basis for formula optimization and individualized treatment strategies. Ultimately, this research will contribute to the modernization and internationalization of TCM by providing evidence-based mechanisms and standardized approaches for Wuling Powder and other TCM formulas in the treatment of functional gastrointestinal disorders.
